# Differences Between the Psychiatric Symptoms of Healthcare Workers Quarantined at Home and in the Hospital After Contact With a Patient With Middle East Respiratory Syndrome

**DOI:** 10.3389/fpsyt.2021.659202

**Published:** 2021-07-16

**Authors:** Su Jeong Seong, Hyung Joon Kim, Kyung Mi Yim, Ji Won Park, Kyung Hoon Son, Yeong Ju Jeon, Jae Yeon Hwang

**Affiliations:** Department of Psychiatry, Hallym University Kangdong Sacred Heart Hospital, Seoul, South Korea

**Keywords:** quarantine, depression, anxiety, acute stress disorder, MERS-CoV

## Abstract

**Objective:** Although quarantine is an effective measure for the prevention of the spread of infectious diseases, it may have negative effects on the mental health of the isolated individual. During the 2015 outbreak of the Middle East Respiratory Syndrome (MERS) in Korea, healthcare workers came in contact with patients with MERS were quarantined either at home or in a hospital ward. In this study, we aimed to compare the psychiatric symptoms of these employees according to the method of quarantine.

**Methods:** All 146 quarantined staff completed self-report questionnaires. Depressive symptoms were measured using the Patient Health Questionnaire-9, anxiety symptoms were assessed using Spielberger's State-Trait Anxiety Inventory, and acute stress disorder (ASD) symptoms were evaluated using the Stanford Acute Stress Reaction Questionnaire.

**Results:** The in-hospital quarantine group had a higher rate of symptoms of depression (*p* < 0.001) and ASD (*p* = 0.014) than the group quarantined at home. Logistic regression analysis showed that respondents quarantined in the hospital (OR = 6.342; 95% CI 1.853–21.708) and those quarantined for longer periods (OR = 1.153, 95% CI = 1.036–1.285) had a higher risk of depressive symptoms.

**Conclusions:** In-hospital quarantine and quarantine for longer periods increase the risk of depressive symptoms. When quarantine measures are taken, certain measures are needed to minimize the risk of psychiatric problems. Appropriate interventions should be implemented if psychiatric problems occur.

## Introduction

The rapid spread of the 2019 Coronavirus disease (COVID-19) resulted in the declaration of a public health emergency of international concern by the World Health Organization on January 30, 2020 (1). Although this pandemic is a crisis that is endangering global health and economy, this is not the first time public health is threatened by the extensive spread of an infectious disease. We have witnessed the epidemics of several infectious diseases, including the severe acute respiratory syndrome (SARS), Ebola, and the Middle East respiratory syndrome (MERS). During the outbreak of the Middle East respiratory syndrome coronavirus (MERS-CoV) infection in Korea from May to December 2015, there were 186 cases of infection and 38 deaths (20.4% of the total number of cases); 16,752 individuals were quarantined due to exposure to persons with MERS (2).

During these epidemics, quarantine was a useful measure widely used to stop the spread of the diseases. It involves the separation and isolation of people who were exposed to a contagious disease (3) to prevent the possible exposure of the general public to contagion. However, the effects of these measures on the psychological well-being of those who experience such restrictions need to be considered. Various adverse psychological outcomes of quarantine and isolation, including depression, mood disorders, anxiety, fear, psychological distress, post-traumatic stress disorder (PTSD), insomnia, irritability, anger, stigmatization, and alcohol use disorder, have been reported in previous studies (4–24). Several studies have shown a high prevalence of depression among isolated subjects. A previous report indicated that up to 77% of patients quarantined because of methicillin-resistant staphylococcus aureus reported depressive symptoms (14). A high prevalence of anxiety has also been consistently reported in several review articles (12). Quarantined individuals reported higher levels of depression and anxiety than those who were not isolated (10, 11, 13, 19, 21, 25–27). Isolation can also cause acute stress, thereby increasing the risk of acute stress disorder (ASD) and PTSD (28). These negative impacts of quarantine and isolation on mental health can continue over a long period. Previous studies have reported an increased risk of alcohol use disorder and depression among quarantined healthcare workers, even 3 years after the SARS outbreak (10, 17).

Hospital staff are exposed to a greater risk of infection and quarantine than the general population during a pandemic of an infectious disease. Of the 2,223 confirmed cases of MERS, 18.7% were healthcare workers. Notably, although the healthcare workers with MERS were younger and had fewer comorbidities than the non-healthcare workers with MERS, they constituted a substantial proportion of the total number of confirmed cases of MERS (29). Hospital staff also tend to experience highly stressful situations such as heavy workload, frequent witnessing of death and trauma, and management of critical medical situations; therefore, their mental health is at greater risk of distress than that of the average individual (4, 30). As a result, healthcare workers in quarantine are thrown into an even more complex and stressful situation. However, factors that influence the mental health of quarantined hospital workers have not yet been thoroughly identified. Specifically, there have been no studies on the effect of the containment method on psychiatric symptoms.

This study aimed to investigate the effect of the quarantine method on psychiatric problems. We hypothesized that staff isolated in a hospital would have more depression, anxiety, and ASD than those who were self-isolated, and that the quarantine method will be a significant predictor of depression, anxiety, and ASD.

## Materials and Methods

### Study Subjects

There was an outbreak of MERS-CoV infection in Korea from May to December 2015. During the outbreak, a hospitalized patient was confirmed to have MERS on June 23, 2015 at Hallym University Kang-dong Sacred Heart Hospital. Hospital staff who were suspected to be in close contact with the patient within 2m had to be mandatorily isolated at home or in the hospital ward until all quarantine measures were lifted on July 7, when there were no more confirmed cases. The psychiatric symptoms of all the 146 employees who were quarantined were evaluated from July 20 to July 24 to help them return to work. Face-to-face counseling and psychiatric treatment were provided for those who suffered significant symptoms. Each subject completed a self-report questionnaire, which included questions on demographic factors such as age, sex, marital status, current cohabitation status, and occupation (medical staff, non-medical staff). Information related to the quarantine, such as route of contact with MERS patients (direct contact, indirect contact), duration of isolation, and type of isolation (in-hospital isolation, self-isolation at home), was also investigated. All completed questionnaires were assigned individual IDs to ensure the confidentiality of the respondents. The data pairing IDs and personally identifiable information were kept privately and were accessible to only the research director. In the present study, the anonymized data collected in 2015 were analyzed retrospectively with the approval of the Institutional Review Board of Kangdong Sacred Heart Hospital.

### Assessment

Depressive symptoms were assessed using the Korean version of the Patient Health Questionnaire-9 (PHQ-9), with a Cronbach's alpha of 0.81 (31). This is a self-rated scale used for the assessment of depressive symptoms. The scale consists of nine items and for each question, the degree of disturbance in daily life is rated from 0 to 3 points; a total of 10 points or more is considered an indication of depression. Ten to 14 points are considered to indicate mild depression, 15–19 points indicate moderate depression, and 20–27 points denote severe depression (32).

Anxiety symptoms were evaluated using the Spielberger's State-Trait Anxiety Inventory (STAI.) This scale consists of 20 items related to state anxiety (SA) and 20 items related to trait anxiety (TA). It is a four-point Likert scale ranging from 1 to 4, depending on the severity of symptoms. A comparison of the SA and TA scores shows whether the cause of the anxiety is an external cause or an individual trait (33). The Korean scales for SA and TA showed an excellent internal consistency (Cronbach's alpha = 0.92 and 0.90, respectively) (34).

The Korean version of the Stanford Acute Stress Reaction Questionnaire (SASRQ) (Cronbach's alpha = 0.98) was used to assess acute stress symptoms (35). The questionnaire consists of 30 items, which have four dimensions, namely: dissociation, re-experience, avoidance, and hyperarousal. Each item is scored from 1 to 5 depending on the severity of symptoms; a score of 3 or more is considered a positive symptom. If the respondent has at least three symptoms in the dissociation area, and more than one symptom in each of the re-experience, avoidance, and hyperarousal areas, he/she considered to have ASD (36).

### Statistical Analysis

We compared the demographic and clinical variables of the in-hospital quarantine group and home-quarantine group using student's *t*-test and Pearson's chi-square test. Furthermore, binary logistic regression analysis was performed to explore the relationships between contributing factors, such as demographic factors and quarantine type and duration, and the occurrence of symptoms of depression, anxiety, and ASD. Odds ratios (ORs) and 95% confidence intervals (CIs) were calculated. The statistical significance level was set at 5% and SPSS 26.0 for Windows was used for all calculations.

## Results

All the 146 staff who were quarantined completed the questionnaires; thus, the participation rate was 100%. The characteristics of the study participants are shown in Table 1. The mean age of the respondents was 33.32 ± 10.78 years; 59 (40.4%) were male, 52 (35.6%) were married, and 105 (71.9%) were living with family. There were 78 (53.4%) medical staff, including 27 doctors and 51 nurses, and 60 (41.1%) non-medical staff, including 12 administrative staff and 48 staff of other occupations (i.e., medical technicians). Regarding the type of exposure to patients with MERS, 66 employees (45.2%) had direct contact during medical treatment, nursing, examination, and patient transfer, whereas 56 (38.3%) had indirect contact in the same space, such as wards and hospital offices; the remaining cases were those who had unknown type of contact. Regarding the type of quarantine, 20 participants (13.7%) were quarantined in the hospital and 123 (84.2%) were self-isolated at home. Two participants were quarantined both at home and in the hospital, and one participant didn't answer the question about the quarantine site. The three participants were excluded from the subsequent analysis where the location of quarantine was in question.

**Table 1:** Sociodemographic characteristics of the respondents according to quarantine method.

	**Total** ** (*n* = 146)**	**In-hospital quarantine** ** (*n* = 20)**	**Self-quarantine at** ** home (*n* = 123)**	**Statistics**	***p***
Sex^a^ (Male)	59 (40.4%)	5 (25.0%)	54 (43.9%)	χ^2^ = 2.536	0.111
Age^b^ (Years)	33.32 ± 10.78	30.60 ± 6.75	33.66 ± 11.13	*t* = −1.192	0.235
Marriage^a^ [*n*, (%)]	52 (35.6%)	8 (40.0%)	44 (35.8%)	χ^2^ = 0.898	0.638
**Living situtation**^**a**^ **[*****n*****, (%)]**				χ^2^ = 3.114	0.211
Alone	31 (21.2%)	5 (25.0%)	26 (21.5%)		
With family	105 (71.9%)	13 (65.0%)	92 (76.0%)		
With others	5 (3.4%)	2 (10.0%)	3 (2.5%)		
**Occupation**^**a**^ **[*****n*****, (%)]**				χ^2^ = 10.668	0.001^*^
Practitioners	78 (53.4%)	18 (90.0%)	60 (50.8%)		
Non-practitioners	60 (41.1%)	2 (10.0%)	58 (49.2%)		
**Contact type**^**a**^ **[*****n*****, (%)]**				χ^2^ = 6.350	0.012^*^
Direct	66 (45.2%)	14 (70.0%)	52 (42.2%)		
Indirect	56 (38.3%)	3 (15.0%)	53 (43.0%)		
Unknown	24 (16.4%)	3 (15.0%)	18 (14.6%)		
Quarantine duration^b^ (day)	13.92 ± 5.62	13.40 ± 3.76	13.18 ± 5.57	*t* = 4.037	0.044^*^

* *p < 0.05*,

a *chi-square analysis was used to test for differences in categorical variables and*

b *t-tests for continuous variables*.

The mean quarantine duration was 13.92 ± 5.62 days, and the longest quarantine period was up to 21 days. Compared to the home-quarantine group, the in-hospital quarantine group had more medical staff (*p* = 0.001), more cases of direct contact (*p* = 0.012), and longer quarantine duration (*p* = 0.044).

Table 2 shows the psychiatric symptoms of the quarantined hospital workers. A total of 38 participants (26%) showed significant depressive symptoms, with a score of 10 or more in the PHQ-9. Twenty-eight (19.1%) of the depressive symptoms experienced by the participants were mild, four (2.7%) were moderate, and six (4.1%) were severe. Regarding anxiety, three (2.1%) respondents reported SA that exceeded the cut-off score of 54 for men and 56 for women. No respondent had a TA score higher than the cut-off scores; this means that the cause of anxiety was external stress, not an internal personality trait. Regarding the SASRQ, 14 (9.5%) participants met the diagnostic criteria for ASD, 23 (15.7%) reported dissociation, 34 (23.2%) reported re-experience, 46 (31.5%) reported avoidance, and 52 (35.6%) participants reported hyperarousal.

**Table 2:** Comparison of psychiatric symptoms according to quarantine method.

	**In-hospital quarantine** ** (*n* = 20)**	**Self-quarantine at** ** home (*n* = 123)**	**Statistics**	***p***
PHQ-9				
Total score^b^	10.35 ± 5.00	5.58 ± 5.37	*t* = 3.711	0.000^*^
**Severity**^**a**^ **[*****n*****, (%)]**			χ^2^ = 25.525	0.000^*^
Not depressed	6 (30.0%)	98 (80.3%)		
Mild	12 (60.0%)	16 (13.0%)			
Moderate	1 (5.0%)	3 (2.4%)		
Severe	1 (5.0%)	5 (4.1%)		
STAI - SA				
Total score^b^	41.55. ± 4.70	40.57 ± 6.81	*t* = 0.617	0.538
**Severity**^**a**^ **[*****n*****, (%)]**			χ^2^ = 0.502	0.778
Not anxious state	20 (100%)	119 (97.5%)		
Mild	0	2 (1.6%)		
Moderate	0	1 (0.8%)		
STAI – TA				
Total score^b^	40.00 ± 4.65	40.20 ± 5.55	t = −0.157	0.875
**Severity**^**a**^ **[*****n*****, (%)]**			
Not anxious trait	20 (100%)	121 (100%)		
SASRQ				
Total score^b^	36.20 ± 23.13	32.86 ± 26.44	*t* = 1.974	0.050^*^
Dissociation^a^ [*n*, (%)]	7 (35.0%)	16 (13.0%)	χ^2^ = 6.164	0.013^*^
Re-experience^a^ [*n*, (%)]	7 (35.0%)	27 (22.0%)	χ^2^ = 1.616	0.204
Avoidance^a^ [*n*, (%)]	8 (40.0%)	38 (30.9%)	χ^2^ = 0.654	0.419
Hyper-arousal^a^ [*n*, (%)]	10 (50.0%)	42 (34.1%)	χ^2^ = 1.868	0.172
**ASD**^**a**^ **[*****n*****, (%)]**			χ^2^ = 6.091	0.014^*^
No ASD	15 (75.0%)	114 (92.7%)		
ASD	5 (25.0%)	9 (7.3%)		

* *p < 0.05*.

a *chi-square analysis was used to test for differences in the categorical variables and*

b *t-tests for continuous variables, PHQ-9, Patient Health Questionnaire-9; STAI, Spielberger's State-Trait Anxiety Inventory; SA, State Anxiety; TA, Trait Anxiety; SASRQ, Stanford Acute Stress Reaction Questionnaire; ASD, acute stress disorder*.

We compared the in-hospital quarantine and home-quarantine groups according to their symptoms of depression, anxiety, and ASD (Table 2). The in-hospital quarantine group showed higher PHQ-9 scores (*p* < 0.001) and had a higher prevalence of depressive symptoms (PHQ-9 score ≥10) (70.0% vs. 19.7%, *p* < 0.001) than the home-quarantine group. The SASRQ results showed that the rates of dissociation symptoms (35.0 vs. 13.0%, *p* = 0.013) and ASD symptoms (25.0 vs. 7.3%, *p* = 0.014) were also significantly higher in the in-hospital quarantine group; however, the difference between the total SASRQ scores of both groups was close to the significance level (*p* = 0.05). Regarding anxiety symptoms, there was no significant difference between the STAI scores of both groups. There was no significant difference in symptoms of depression, anxiety, and ASD when the participants were compared according to factors other than the type of isolation.

Logistic regression analysis was performed to identify factors associated with symptoms of depression and ASD. Regarding anxiety, the number of subjects with STAI scores that exceeded the cut-off point was too small to be analyzed. Four respondents who did not have the necessary information were excluded from the analysis. Type of isolation (*p* = 0.003) and quarantine duration (*p* = 0.009) were significantly associated with depressive symptoms measured using PHQ-9 score (Table 3). The in-hospital quarantine group had a higher risk of depressive symptoms than the home-quarantine group (OR = 6.342; 95% CI 1.853–21.708). Each day of isolation increased the risk of depressive symptom 1.153-fold (OR = 1.153; 95% CI 1.036–1.285). No variables were significantly associated with any symptom of ASD measured with the SASRQ.

**Table 3:** Variables that affect presence of depressive symptoms.

**Variables**	**ß**	**s.e**.	**Wald**	**OR (95% CI)**	***p***
Age^a^ (year)	0.042	0.032	1.731	1.043 (0.979–1.111)	0.188
**Sex**^**b**^					
Male (base = female)	0.466	0.509	0.838	1.594 (0.588–4.324)	0.360
**Marriage**^**b**^					
Single (base = married)	−0.248	0.642	0.149	0.781 (0.222–2.748)	0.700
**Living situation**^**b**^					
Alone (base = with family)	−0.767	0.631	1.478	0.465 (0.135–1.599)	0.224
**Occupation**^**b**^					
Practitioner (base = non-practitioner)	0.598	0.535	1.249	1.819 (0.637–5.197)	0.264
**Contact type**^**b**^					
Direct contact (base = indirect)	−0.424	0.765	0.307	0.655 (0.146–2.931)	0.580
Quarantine duration^a^ (day)	0.143	0.055	6.744	1.153 (1.036-1.285)	0.009^**^
**Quarantine method**^**b**^					
In-hospital (base = at home)	1.847	0.628	8.655	6.342 (1.853–21.708)	0.003^**^

** *p < 0.01, a, continuous variables; b, categorical variables; OR, odds ratio; CI, confidence interval. Logistic regression was used to evaluate the association between depression (PHQ-9 score ≥10) and variables. All variables are shown in Table 3*.

## Discussion

The present study revealed that a large number of quarantined staff showed symptoms of depression (26%) and ASD (9.5%). In previous studies, the prevalence of psychiatric symptoms among quarantined individuals varied greatly depending on the characteristics of the participants, hospitalization, situation and causes of quarantine, scales used for assessment, and the timing of symptom measurement. The prevalence of depressive symptoms ranged from 3% (5) to 77% (14). In the present study, the prevalence of depressive symptom (26%) was lower than the prevalence (38.8%) reported 3 years later for a group of health workers who were quarantined during the SARS epidemic (10), and higher than the prevalence (15.1%) reported for patients on hemodialysis who were quarantined in the hospital during the MERS outbreak (7). This difference may be due to differences in the tools used to measure depressive symptoms or differences in the mortality and infectivity of the disease. Another plausible reason may be because hospital workers experience greater stigmatization than the general population (30). Zhou et al. (37) reported that frontline medical staff had a greater risk for psychological disturbances, including depression and anxiety, than the general population did during the early phase of the COVID-19 pandemic. They also found that workload significantly affected the psychological disturbances of the medical staff. However, we did not include variables regarding the working environment, such as workload. In our study, the hospital staff had not experienced a sustained increase in workload, as the exposure was made by only one case and short-lived. Therefore, symptoms of anxiety and depression were more likely to be attributable to the quarantine measures.

The prevalence of significant depressive symptoms in the hospital staff was 26%, significantly higher than the 12-month prevalence of depression (0.3–10%) in the general population (38), although the figure was drawn from PHQ-9, not from a psychiatrist's diagnosis or structured interview. Thus, the actual prevalence of major depressive disorder may have been lower. Nevertheless, we speculate that a significant proportion of the hospital staff experienced depression, unlike before, after the exposure to the patient with MERS as we observed that the quarantine measures had direct effects on significant depressive symptoms.

The percentage of participants in the present study that complained of anxiety symptoms was small (2.1%) compared to those of previous studies, which reported values that ranged from 7.6% (16) to 45.8% (13). Our study also showed a lower rate of ASD (9.5%) than the 17% (28) and 28.9% (9) reported for healthcare workers quarantined during the SARS epidemic. However, the prevalence of ASD in the present study is similar to the 10% reported for hospital workers 3 years after the SARS outbreak (18). This may be due to the timing of the assessment. A study by Chong et al. showed that the rate of anxiety and ASD decreased, whereas the rate of depression increased 3 weeks after the epidemic subsided (39). Jeong et al. also reported diminished anxiety symptoms 4–6 months after quarantine (16). These results suggest that symptom profiles can change depending on the time of the investigation. Since the surveys analyzed in the present study were carried out about a month after the start of isolation, less anxiety and PTSD symptoms and more depressive symptoms were likely to be reported.

The symptoms of depression and ASD were more common and severe among the participants quarantined in the hospital than among those quarantined at home. The risk of depressive symptoms was also higher among the staff quarantined in the hospital than among those quarantined at home and those who were quarantined for longer periods. These results suggest that methods of quarantine can affect the frequency and severity of the psychological adverse effects of isolation. Regarding the duration of the isolation period, several studies reported that the longer the isolation period, the higher the risk of having psychological problems such as PTSD (9, 30), emotional exhaustion, and anger (40). On the other hand, there have been reports that the duration of the isolation period is not associated with depression (14, 41) or PTSD (5, 42). Tarzi et al. explained that it is difficult to discuss the association between the duration of isolation and depression because the duration of quarantine was more than 2 weeks for all subjects in their study (14). Cho et al. reported that for some participants, a short quarantine period meant a delayed start of isolation due to poor compliance (42). In the study by Tang, the isolation period was analyzed as a non-continuous variable based on randomly divided intervals (1, 2, and 4 weeks) instead of a continuous variable (5). Regarding the quarantine location, no previous studies have directly compared the differences in psychiatric symptoms according to quarantine locations. In previous studies, loneliness was strongly associated with depression, anxiety, and PTSD (43). Without psychological support, symptoms of depression and anxiety become more severe (8). Notably, social networking activities (16) and social support (44) have been shown to lower the risk of anxiety and PTSD symptoms. Compared to self-isolation at home, social support when quarantined in a hospital and separated from family members is likely not enough to prevent depression. Therefore, a lack of social support could contribute to an increased risk of depressive symptoms.

To the best of our knowledge, this is the first study in which the psychiatric symptoms of hospital staff isolated during an epidemic were compared according to quarantine methods. Since only one confirmed case was admitted to the hospital, it was easy to track the path of infection to ensure that all employees who had contact with the contagion were quarantined. All those who were quarantined completed the questionnaire. Therefore, the selection bias of the study subjects was minimized.

However, this study has several limitations. First, since this study was conducted in a general hospital, there may be limitations regarding generalizing the findings to other situations. Second, due to the cross-sectional nature of the investigation, it was difficult to consider the effect of time, which has an important effect on the onset and course of symptoms after acute stress. Third, our study didn't include other variables that can affect the mental health of hospital staff, like preexisting health-related problems and workload. In addition, the long-term effects of quarantine on the staff were not studied. Considering that psychiatric problems were reported in previous studies even three years after implementation of quarantine measures, long-term sequelae could remain after the isolation due to MERS.

In conclusion, we found that the prevalence rates of the symptoms of depression and ASD among quarantined healthcare workers are high (26 and 9.5%), and are higher in the in-hospital quarantine group than in the home-quarantined group. In addition, the likelihood of having depressive symptoms increased when quarantined in a hospital and when the duration of isolation became longer. This result suggests that the method of quarantine needs to be modified to minimize its adverse psychological effect on the isolated individual. The quarantine site or environment should be set up to lower the risk of psychological side effects. In addition, the duration of isolation should be the minimum required. If in-hospital and longer isolation is needed, proper preventive measures must be implemented, and psychological support must be provided. During and after isolation, psychiatric evaluation and intervention must be carried out more actively. Since quarantined hospital staff are at higher risk of showing the symptoms of depression and ASD, quarantine protocol that can help reduce stress and timely intervention for symptoms that occur need to be in place at each general hospital. Further studies are needed to determine which interventions are effective in situations of isolation caused by infectious diseases.

## Data Availability Statement

The data that support the findings of this study are available on request to the corresponding author. The data are not publicly available due to privacy concerns.

## Ethics Statement

The studies involving human participants were reviewed and approved by the Institutional Review Board of Kangdong Sacred Heart Hospital. The ethics committee waived the requirement of written informed consent for participation.

## Author Contributions

KY contributed to the design of the study and HK, KY, JP, KS, and YJ contributed to the acquisition of data. HK and SS conducted statistical analyses and SS wrote the draft. HK and YJ provided significant input on the manuscript. JH contributed to the design of the study, supervision of data collection, and provision of significant input on the manuscript. All the authors read and approved the final manuscript.

## Conflict of Interest

The authors declare that the research was conducted in the absence of any commercial or financial relationships that could be construed as a potential conflict of interest.
