# Supplementary material for: Differences Between the Psychiatric Symptoms of Healthcare Workers Quarantined at Home and in the Hospital After Contact With a Patient With Middle East Respiratory Syndrome
Source: Front Psychiatry. 2021 Jul 16;12:659202. doi: 10.3389/fpsyt.2021.659202 (PMC8322759; doi:10.3389/fpsyt.2021.659202)
Supplement: Supplementary file 1 [file Data_Sheet_1.PDF]

Supplementary Material

Supplementary Table 1. Variables that affect presence of acute stress disorder symptoms

Variables	ß	s.e.	Wald	OR (95% CI)	p
Age[a]	-0.001	0.054	0.000	0.990 (0.899-1.111)	0.990
Sex[b]					
Male (base=female)	-0.218	0.703	0.096	0.804 (0.203-3.194)	0.757
Marriage[b]					
Single (base=married)	-0.765	1.016	0.568	0.465 (0.064-3.406)	0.451
Living situation[b]					
Alone (base= with family)	0.778	0.889	0.766	2.177 (0.381-12.429)	0.381
Occupation[b]					
Practitioner (base=non-practitioner)	-0.052	0.820	0.004	0.950 (0.190-4.740)	0.950
Contact type[b]					
Direct contact (base=indirect)	0.762	0.786	0.940	2.144 (0.459-10.011)	0.332
Quarantine duration[a] (day)	0.010	0.075	0.017	1.010 (0.872-1.170)	0.895
Quarantine method[b]					
In-hospital (base=at home)	1.376	0.802	2.942	3.959 (0.822-19.067)	0.086

**p<0.01, a: continuous variables, b: categorical variables. OR: odds ratio, CI: confidence interval. Logistic regression was used to evaluate the association between depressive symptoms (PHQ-9 score ≥10) and variables.

Supplementary Table 1. Variables that affect presence of acute stress disorder symptoms

**p<0.01, a: continuous variables, b: categorical variables. OR: odds ratio, CI: confidence interval. Logistic regression was used to evaluate the association between depressive symptoms (PHQ-9 score ≥10) and variables.
